# A Survey of the Knowledge of Venous Thromboembolism Prophylaxis among the Medical Staff of Intensive Care Units in North China

**DOI:** 10.1371/journal.pone.0139162

**Published:** 2015-09-29

**Authors:** Xiao Tang, Bing Sun, Yuanhua Yang, Zhaohui Tong

**Affiliations:** 1 Department of Respiratory and Critical Care Medicine, Beijing Chao-Yang Hospital, Capital Medical University, Beijing, China; 2 Beijing Institute of Respiratory Medicine, Beijing, China; The Ohio State University, UNITED STATES

## Abstract

**Background:**

Guideline concordance for venous thromboembolism (VTE) prophylaxis in critically ill patients in intensive care units (ICUs) varies across different countries.

**Objective:**

To explore how the medical staff of ICUs in China comprehend and practice VTE prophylaxis.

**Method:**

Questionnaires comprising 39 questions and including 4 dimensions of thromboprophylaxis were administered in ICUs in North China.

**Results:**

In all, 52 ICUs at 23 tertiary hospitals in 7 Chinese provinces and municipalities were surveyed. A total of 2500 questionnaires were sent, and 1861 were returned, corresponding to a response rate of approximately 74.4%. Of all surveyed medical staff, 36.5% of physicians and 22.2% of nurses were aware of the guidelines in China, and 19.0% of physicians and 9.5% of nurses comprehended the 9^th^ edition of the guidelines of the American College of Chest Physicians (ACCP). Additionally, 37.6% of the medical staff chose a prophylaxis method based on the related guidelines, and 10.3% could demonstrate the exact indication for mechanical pattern application. Worries about skin injury, difficulty with removal and discomfort during mechanical thromboprophylaxis were cited by more than 30% of nurses, which was significantly more frequent than for physicians (graduated compression stockings: 54.3% VS 34.1%, 60.7% VS 49%, and 59.4% VS 54%, *p* = 0.000; intermittent pneumatic compression: 31% VS 22.2%, 19.2% VS 13.9%, and 37.8% VS 27.2%, *p* = 0.000).

**Conclusions and Relevance:**

The knowledge of VTE prophylaxis among the medical staff of ICUs in North China remains limited, which may lead to a lack of standardization of VTE prophylaxis. Strengthened, standardized training may help medical staff to improve their comprehension of the relevant guidelines and may finally reduce the occurrence of VTE in ICUs and improve the prognosis of critically ill patients with VTE.

## Introduction

Venous thromboembolism (VTE) is a common medical condition that manifests as deep vein thrombosis (DVT) and/or pulmonary embolism (PE). Critically ill patients in the intensive care unit (ICU) are at high risk for VTE because of their specific conditions, such as immobilization, post-operative status, sepsis, mechanical ventilation and central venous catheter use. A systematic review[1] showed that the incidence of DVT ranged from 13–31% without prophylaxis. Moreover, Ribic’s team[2] found that the frequency of VTE in patients receiving low-molecular-weight heparin (LMWH) ranged from 5.1–15.5%.

The American College of Chest Physicians (ACCP) Antithrombotic Therapy and Prevention of Thrombosis guidelines, in which recommendations for VTE prophylaxis in critically ill patients are provided, are updated every 2–4 years[3]. In 2009, the Chinese Society of Critical Care Medicine also issued a guideline about DVT prophylaxis in critically ill patients in ICUs[4]. Although many studies have shown guideline concordance for thromboprophylaxis in ICUs in the West[5],[6], Few studies have examined VTE prophylaxis in China. The aim of this research was to explore how the medical staff of ICUs in China comprehend and practice VTE prophylaxis.

## Materials and Methods

### 1 Subjects

This survey began in September 2014 and was completed in January 2015. The ICUs involved in this survey were at tertiary hospitals in North China. These ICUs included surgical, medical and other specialized ICUs. Participants included the physicians and nurses working in these ICUs. This was a paper questionnaire-based survey. In particular, questionnaires were sent to the ICUs and were returned after completion. Each site had a director who had been trained to send the questionnaires to individual physicians and nurses and to collect the questionnaires after they were finished. All of the participants were asked to objectively and honestly answer the questions. The survey was anonymous, and no names or other identifying data were recorded.

### 2 Survey design

The questionnaire was co-designed by specialists who are experts in the fields of VTE and critical care medicine. The survey comprised 39 questions covering 4 dimensions: (1) general information on the participant, (2) awareness of relevant guidelines that address VTE prophylaxis in critically ill patients in ICUs, (3) the practice pattern of VTE prophylaxis in the participant’s ICU, and (4) concerns regarding pharmacological and mechanical patterns during VTE prophylaxis. Before being widely distributed, the survey was pilot tested on a small number of faculty members within our respiratory ICU division for review and comment.

### 3 Data analysis

EpiData 3.1 was applied to establish a database. The completed questionnaires were entered twice into the database and checked for consistency and accuracy. The verified data were then imported into SPSS 19.0 for Windows to generate appropriate descriptive statistics. Additionally, several statistical comparisons were performed among binary variables using Pearson’s χ^2^ test at a 95% significance level.

### 4 Research ethics

This survey study was reviewed and approved by the ethics committee of Beijing Chao-Yang Hospital of China, which waived the need for informed consent.

## Results

### 1 General information

Cronbach’s alpha for this survey was 0.773. Factor analysis yielded the Kaiser-Meyer-Olkin coefficient, which was 0.751.

From September 2014 to January 2015, 52 ICUs at 23 tertiary hospitals in 7 provinces and municipalities in North China participated in the survey. A total of 2500 questionnaires were sent, and 1861 were returned, which corresponded to approximately 74.4% of all of the medical staff working at the ICUs involved in this survey.

The 52 ICUs included 21 medical ICUs, 7 surgical ICUs and 24 general ICUs. The 1681 participants involved in this survey comprised 564 physicians and 1117 nurses (Table 1).

**Table 1:** General information about the medical staff involved in the survey.

**Item**	Classification		Number of ICUs	Number of physicians	Number of nurses
**Working years**		0–3 years		284(50.4%)	404(36.2%)
		3–5 years		114(20.2%)	241(21.6%)
		5–10 years		90(16.0%)	302(27%)
		>10 years		73(12.9)	169(15.1%)
**ICUs**	Medical ICUs	Medical ICUs	1	41(2.4%)	90(5.3%)
		Respiratory ICUs	16	162(9.6%)	258(15.3%)
		Neurology ICUs	4	30(1.8%)	58(3.5%)
		Total	21	234(41.5&)	406(36.3%)
	Surgical ICUs	Surgical ICUs	5	60(3.6%)	100(5.9%)
		Cardiac surgery ICUs	1	5(0.3%)	30(1.8%)
		Neurosurgical ICUs	1	14(0.8%)	22(1.5%)
		Total	7	78(13.8%)	149(13.3%)
	General ICUs	Emergency ICUs	9	101(6.0%)	172(10.2%)
		General ICUs	15	144(8.6%)	379(22.5%)
		Total	24	252(44.7%)	562(50.3%)
**Titles**		Resident physician		270(47.9%)	
		Attending physician		203(36.0%)	
		Associate chief physician		62(11%)	
		Chief physician		26(4.6%)	
		Nurse			484(43.3%)
		Primary nurse			510(45.7)
		Nurse-in-charge			102(9.1%)
		Co-chief superintendent nurse			14(1.3%)
		Chief superintendent nurse			6(0.5%)
**Total**			52	564(33.6%)	1117(66.4%)

### 2 Comprehension of the relevant content of VTE prophylaxis in critically ill patients

#### 2.1 Self-evaluation of the medical staff regarding comprehension of VTE prophylaxis

A total of 66% of physicians and 83.6% of nurses thought that they generally understood VTE prophylaxis, and 37.1% of physicians and 8.6% of nurses indicated that they had mastered the knowledge well. The self- evaluation of the physicians was significantly higher than that of the nurses (*p* = 0.000) (Table 2).

**Table 2:** Self-evaluation of comprehension of VTE prophylaxis.

Post		None	General	Extremely	*p* ^*c*^
**Physicians**	Resident physician	9(0.5%)	218(13.0%)	43(2.6%)	
	Attending physician	2(0.1%)	120(7.1%)	81(4.8%)	
	Associate chief physician	0(0%)	27(1.6%)	35(2.1%)	
	Chief physician	1(0.1%)	5(0.3%)	20(1.2%)	
	**Total number of physicians** ^**a**^	**13(2.3%)**	**372(66%)**	**179(37.1%)**	
**Nurses**	Nurses	48(2.9%)	403(24%)	29(1.7%)	
	Primary nurse	29(1.7%)	434(25.8%)	45(2.7%)	
	Nurse-in-charge	3(0.2%)	84(5.0%)	15(0.9%)	
	Co-chief superintendent nurse	1(0.1%)	9(0.5%)	4(0.2%)	
	Chief superintendent nurse	0(0%)	3(0.2%)	3(0.2%)	
	**Total number of nurses** ^**b**^	**81(7.3%)**	**934(83.6%)**	**96(8.6%)**	
**Total**		**94(5.6%)**	**1306(77.7%)**	**275(16.4%)**	0.000

Comments

a- The percentage of the total number of physicians

b- The percentage of the total number of physicians

c- Comparison between physicians and nurses in the self-evaluation of comprehension of VTE prophylaxis

#### 2.2 Necessity and practice of VTE prophylaxis in critically ill patients in ICUs

A total of 96.6% of physicians and 96.2% of nurses thought that it was necessary to practice VTE prophylaxis. In total, 74.3% of physicians and 65.5% of nurses believed that both pharmacological and mechanical methods should be used, whereas 6.2% of physicians and 3.2% of nurses believed in primarily using a pharmacological method (Table 3).

**Table 3:** How to perform VTE prophylaxis.

	Professed pattern			Actual pattern			*p* *
	Physicians	Nurses	Total	Physicians	Nurses	Total	
**Mainly pharmacological**	35(6.2%)	36(3.2%)	71(4.2%)	83(14.7%)	117(10.5%)	200(11.9%)	
**Mainly mechanical**	106(18.8)	328(29.4%)	434(25.8%)	113(23.2%)	331(29.6%)	462(27.5%)	
**Combined pharmacological and mechanical prophylaxis**	419(74.3%)	732(65.5%)	1151(65.8%)	334(59.2%)	611(54.7%)	945(56.2%)	
**Unknown**	2(0.4%)	13(1.2%)	15(0.9%)	9(1.6%)	39(3.5%)	48(2.9%)	
**Other**	0(0%)	2(0.2%)	2(0.1%)	6(1.1%)	9(0.8%)	15(0.9%)	0.000

Comment

*- Comparison of the distribution between the professed pattern and the actual pattern

#### 2.3 Awareness of the relevant guidelines addressing VTE prophylaxis in critically ill patients in ICUs

A total of 36.5% of physicians and 22.2% of nurses were aware of the guidelines in China.

In 2012, the 9th edition of the ACCP guidelines[3] was updated; 19.0% of physicians and 9.5% of nurses comprehended these guidelines.

A total of 60.6% of physicians and 49.7% of nurses had a general understanding of VTE risk assessment. However, 25.2% of physicians and 42.5% of nurses did not understand this concept.

Additionally, 45.5% of the medical staff of the ICUs stated that they assessed the risk of VTE in patients. In contrast, 20.9% never did, and 28.3% did not know how to evaluate the risk of VTE.

#### 2.4 VTE risk in ICU patients

The risk of VTE among ICU patients is high. The results showed that more than 60% of the medical staff thought that a varicose vein in a lower extremity (75.9%), immobility (71.6%), post-operative status (68%), and VTE history (61%) were risk factors for VTE in ICU patients. Other risk factors noted were chronic illness (55.6%) and deep venous catheters (49%). Less than 40% of the medical staff considered sedation (37.7%), mechanical ventilation (35.8%), cancer (34.5%), vasopressor use (27.1%) and acute illness (25.8%) as risk factors for VTE in ICU patients. Finally, oral contraceptive use, hematological diseases, advanced age, pregnancy and continuous renal replacement therapy were considered VTE risk factors by 2.7% of the medical staff.

#### 2.5 Thrombosis and bleeding: which is more terrifying?

The answers to this question significantly differed between surgical, medical and general ICUs (*p* = 0.000). A total of 55.5% of the medical staff in respiratory ICUs, neurology ICUs and medical ICUs worried more about VTE events, whereas 60.1% of the staff in surgical ICUs worried more about hemorrhage. In contrast, the attitudes of general ICU medical staff toward the two aspects were equally split (49.7% for thrombosis VS 50.3% for bleeding).

Significantly different responses were found in our further investigation of the subspecialty ICUs (*p* = 0.010). More specifically, the medical staff of cardiac surgery ICUs and neurosurgical ICUs feared hemorrhage the most (71.4% and 60%, respectively), whereas the staff of medical ICUs (57.1%) worried the most about thrombotic events, as did the staff of respiratory ICUs (55.4%) and neurology ICUs (53.6%).

### 3 Practice of VTE prophylaxis in ICUs

#### 3.1 Patterns of the VTE prophylaxis practiced in ICUs

A total of 40.6% of the medical staff practiced VTE prophylaxis according to the situation and used conventional therapy in the ICUs, 37.6% chose prophylaxis methods based on the related guidelines, and 9.8% chose methods based on their experience. Nevertheless, 5.1% of the medical staff had no idea how to practice VTE prophylaxis.

total of 56.2% of the medical staff chose to use both pharmacological and mechanical methods to practice VTE prophylaxis, whereas 11.9% of the medical staff selected a pharmacological method only. The actual pattern that they chose to practice was different from what they professed (*p* = 0.000) (Table 3).

**Fig 1:**
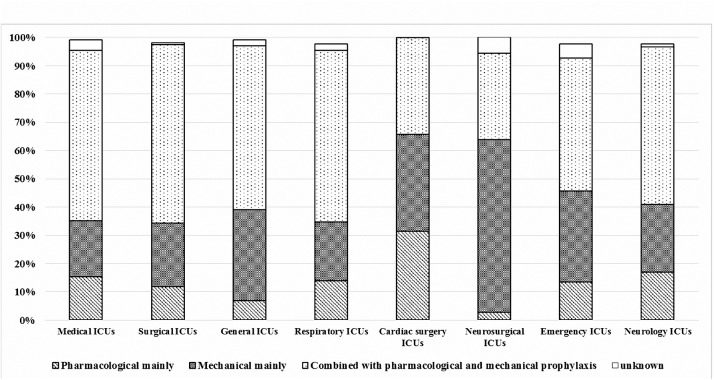
Tendencies in VTE prophylaxis pattern choices in various ICUs.

The medical staff of various subspecialty ICUs chose different methods for VTE prophylaxis (*p* = 0.001). In particular, the medical staff of neurosurgical ICUs (61.1%) preferred to use only mechanical methods. In contrast, the physicians and nurses of surgical ICUs (63.1%), medical ICUs (60.3%) and respiratory ICUs (60.7%) chose a combination of both pharmacological and mechanical approaches (Fig 1).

#### 3.2 Pharmacological VTE prophylaxis methods

A total of 83.8% of the medical staff chose LMWH, and 12.3% selected unfractionated heparin (UFH). In all, 17.8% and 17.5% of the medical staff used warfarin and aspirin, respectively, and 1.8% chose other drugs, including new factor Xa inhibitors, fondaparinux, clopidogrel, and ticagrelor, among others.

emorrhage was undoubtedly the most anxiety-provoking event. In performing pharmacological VTE prophylaxis, 81.6% of the medical staff worried about bleeding, 76.2% were afraid of thrombus shedding, and 33.9% doubted the effect of the pharmacological approach. Moreover, 32.6% worried about liver and kidney function, and 17.7% worried about allergy.

#### 3.3 Mechanical VTE prophylaxis

A total of 10.3% of the medical staff knew the appropriate indications for mechanical VTE prophylaxis, which include bleeding, a high risk of major bleeding, a non-hemorrhagic contraindication for pharmacological prophylaxis and a contraindication for combined pharmacological and mechanical prophylaxis. A total of 51.9% of the medical staff believed that a mechanical approach must be combined with pharmacological prophylaxis, and 28.3% believed that mechanical prophylaxis could be practiced at any time. Additionally, 5.1% had no idea when mechanical prophylaxis could be used.

ntermittent pneumatic compression (IPC) and graduated compression stockings (GCS) are the common measures used in mechanical VTE prophylaxis in ICUs. A total of 35.3% of the medical staff thought that combining IPC and GCS was necessary to practice mechanical prophylaxis, 30.1% were uncertain as to whether the approaches needed to be combined, and 15.7% deemed the combination unnecessary.

The proportion of the medical staff considering GCS to be effective was approximately 46.9%, and approximately 52% considered IPC to be useful. However, 38.9% of the medical staff were uncertain about the effect of GCS, and 27.6% doubted the efficacy of IPC. No significant difference was found between the medical staff’s perspectives on the efficacy of IPC and GCS (*p* = 0.654).

An apparent distinction was found between physicians and nurses regarding anxiety toward mechanical VTE prophylaxis.

**Fig 2:**
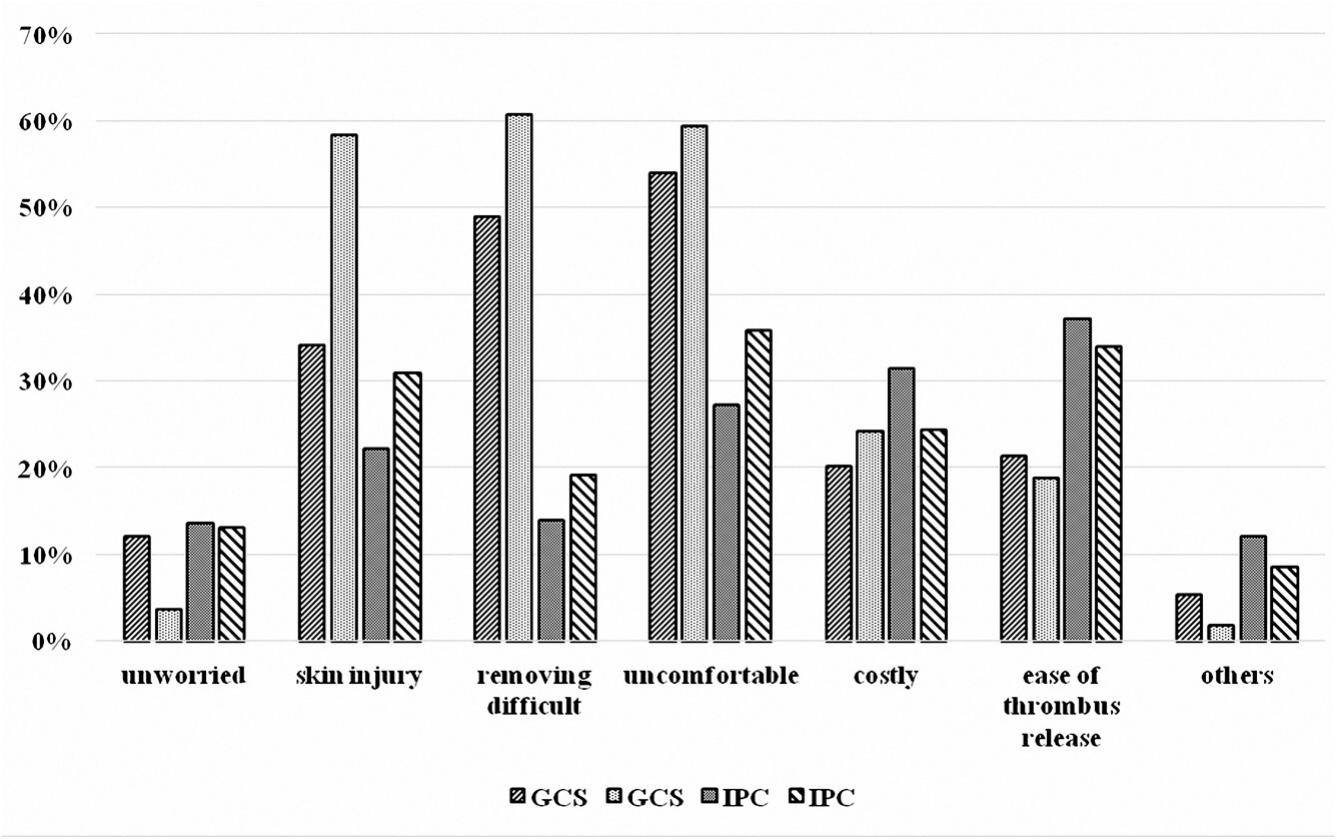
Concerns about mechanical prophylaxis among the medical staff.

A total of 12.1% of physicians expressed that they do not hesitate to use GCS, but only 3.6% nurses did not worry about this method (*p* = 0.001). The differences between physicians and nurses were evidenced by discrepancies in the following concerns: that GCS easily induce skin injury (34.1% VS 54.3%, *p* = 0.000), that GCS can be difficult to remove (49% VS 60.7%, *p* = 0.000), and that GCS cause patient discomfort (54% VS 59.4%, *p* = 0.003). A few nurses also said that GCS increased their burden of work, given that they had to remove the GCS to check the condition of the skin at the start of their shift because GCS increase the risk of ulcers in the lower legs and because GCS can be contaminated by stool, among other reasons (Fig 2).

Regarding IPC, 13.5% of physicians and 13% of nurses indicated no anxiety, but an obvious difference was found between nurses and physicians regarding concerns about skin injury (22.2% VS 31%, *p* = 0.000), difficulty removing (13.9% VS 19.2%, *p* = 0.001) and patient discomfort (27.2% VS 37.8%, *p* = 0.000). Other concerns included the ease of cross-infection because the leg sleeves of IPC are reprocessed after sterilization, a lack of nurses, patient reluctance, and the expensive consumptive material required for IPC, among other concerns (Fig 2).

However, comparing anxiety toward GCS and IPC revealed that the worries about GCS causing skin injury (49.9% VS 27.9%) as well as difficulty removing GCS (57.1% VS 17.3%) and discomfort (57.3% VS 32.7%) were greater than the anxiety toward IPC (*p* = 0.000). Regarding cost (22.7% VS 26.5%) and the ease of thrombus release (19.5% VS 34.9%), there were fewer concerns about IPC than about GCS (*p* = 0.000) (Fig 2).

## Discussion

Thrombosis prophylaxis has been receiving increasing attention from physicians and specialists in ICUs. However, our survey results revealed deficient understanding of thrombosis prophylaxis among the medical staff of ICUs in North China. This lack of knowledge may lead to a lack of standardization of thromboprophylaxis in ICUs. First, knowledge of the relevant guidelines was insufficient. Second, the physicians rarely assessed the risk of thrombosis in patients in the ICUs and arbitrarily practiced thromboprophylaxis; few doctors could execute standard measures. Finally, anxiety regarding the side effects of pharmacological prophylaxis and doubts and anxiety about mechanical approaches limited generalized prophylaxis use.

The conditions of ICU patients are severe and complex; thus, physicians must pay close attention to organ support and therefore often ignore VTE events. Nevertheless, as VTE has been increasingly noted in recent years and as related guidelines have been established, more physicians are considering VTE and VTE prophylaxis. Certain studies have indicated that the incidence of VTE decreased from 13–31% to 5.1–15.5% with the use of VTE prevention[1, 2]. Research from Australia and New Zealand[7] showed that omitting thromboprophylaxis within the first 24 h of ICU admission without obvious reasons was associated with an increased risk of mortality in critically ill adult patients. Research from South America[6] and Romania[8] showed that VTE prophylaxis was also in accordance with the recommendations of the national guidelines. However, a survey from Austria[5] showed that VTE prophylaxis guideline adherence was only 40%. Therefore, the practice of thromboprophylaxis varies widely across countries.

The use of VTE prophylaxis in mainland China has lagged behind that in the West. Based on the results of the survey described here, although the concept of thromboprophylaxis was developed nearly 20 years ago, the perception of VTE prophylaxis among the medical staff of Chinese ICUs is still deficient. In particular, in our study, 60% of the medical staff were not aware of the VTE prophylaxis guidelines in China or abroad. In addition, less than half of the medical staff assessed the risk of VTE in ICU patients, and more than 50% of the medical staff chose nonstandard approaches. VTE prophylaxis in ICUs in China obviously lacks standardization, which is most likely due to the lack of knowledge of relevant guidelines. Thus, training as well as dissemination of these guidelines must be strengthened.

Recently, certain research has indicated the effect of mechanical thromboprophylaxis in critically ill patients in ICUs. According to a systematic review[9], limited evidence suggests that the use of compressive and pneumatic devices does not yield significantly different results compared with no treatment. Research by Lilly et al.[10] also indicated that the mortality risk of those receiving mechanical device prophylaxis was not lower than that of patients without VTE prophylaxis and that patients managed with prophylactic anticoagulation therapy had a significantly lower risk of death than those receiving only mechanical device prophylaxis. A study comparing IPC and GCS[11] showed that the use of IPC was associated with a significantly lower VTE incidence compared with no mechanical thromboprophylaxis but that GCS use was not associated with a decreased VTE incidence. The role of mechanical thromboprophylaxis in VTE prevention in ICUs thus remains uncertain. According to our survey, the medical staff doubted the efficacy of mechanical prophylaxis, and especially GCS. Nevertheless, GCS and/or IPC use in patients who are bleeding or at high risk for major bleeding remains mentioned in VTE prophylaxis guidelines[3, 12, 13]. If mechanical thromboprophylaxis is to be widely used in VTE prevention, however, further evidence of its efficacy must be demonstrated.

In addition to a lack of efficacy evidence, another limitation of mechanical thromboprophylaxis may be hesitancy to use it in clinical practice. In our survey, worries regarding skin injury, difficulty removing GCS and the discomfort of mechanical thromboprophylaxis were expressed by more than 50% of nurses; similar findings were found in a study from Korea[14]. Because nurses provide bedside care, the demands of their work are greater than those for physicians, which may explain the increased anxiety of nurses. Therefore, avoiding or reducing these issues may help to increase the popularity of mechanical thromboprophylaxis.

This study has certain limitations. First, because this research was conducted in North China, the findings may not be generalizable to medical staff in ICUs elsewhere in China. Second, fewer surgical ICUs than medical ICUs and general ICUs were involved in this survey, and the distribution of subspecialty ICUs was asymmetrical, which may have introduced bias into the results. Third, the questionnaire asked many subjective questions, impacting the objectivity of the survey. Nonetheless, we still think that this survey demonstrates the status of VTE prophylaxis in China. A standardized VTE prophylaxis strategy should be established among the medical staff of ICUs to reduce the occurrence of VTE in the future.

## Conclusion

The knowledge of VTE prophylaxis among the medical staff of ICUs in North China is deficient, which may lead to a lack of standardization of VTE prophylaxis. Strengthened and standard training may help medical staff to improve their understanding of relevant guidelines. Clinical research and guidelines that are consistent with Chinese culture and that have been developed in related fields could also enhance the acceptability and execution of VTE prophylaxis. These approaches may finally reduce the occurrence of VTE in ICUs and ameliorate the prognosis of critically ill patients with VTE.

## Supporting Information

S1 Text Relevant data underlying the findings described in manuscript. (SAV)
